# Association Between Obesity Indices and Heart Rate Variability: A Cross‐Sectional Study in Asian Young College Students

**DOI:** 10.1155/crp/5718188

**Published:** 2026-06-13

**Authors:** Jun Wang, Xuechun Ding, Juan Wang, Juan Wu, Dongdong Zhu, Juxia Chen, Zhixiang Peng

**Affiliations:** ^1^ Department of Medicine, Anqing Medical College, Anqing, Anhui, China

**Keywords:** autonomic function, body fat percentage, heart rate variability, obesity indices, young adults

## Abstract

**Objective:**

This study aimed to evaluate the associations of various traditional and novel obesity indices with heart rate variability (HRV) in Asian young college students, assessing their relative importance and potential sex differences.

**Methods:**

In this cross‐sectional study of 3180 Asian young college students (mean age 19.6 years), we measured eight obesity indices including body mass index (BMI), waist circumference (WC), waist‐to‐height ratio (WHtR), and body fat percentage (BFP). HRV was assessed from a 5‐min resting electrocardiogram and analyzed across three autonomic domains derived from principal component analysis. We employed sex‐stratified linear models followed by a multimodel approach (best subset selection, relative importance analysis, and elastic net regression) to control for collinearity. All analyses were adjusted for age, heart rate, physical activity, and measurement time.

**Results:**

BFP demonstrated the strongest associations with HRV parameters, showing significant negative correlation with SDNN (*β* = −1.76) and positive correlations with LF/HF (*β* = 0.123) and LFnorm (*β* = 1.84) (all FDR *p* < 0.001). Relative importance analysis showed that BFP contributed most to predicting LF/HF (38.1%), significantly exceeding WC (17.5%) and BMI (13.9%). The addition of BMI to BFP was associated with improved prediction for LF/HF and LFnorm (Δ*R* ^2^ > 0.03). Subgroup analysis showed that overweight/obese individuals with elevated BFP had significantly impaired HRV across all indices, while those with normal BFP showed reduced SDNN only. ROC analysis gave a BFP cutoff of 27.3% for identifying low HRV risk. No significant sex interactions were observed.

**Conclusion:**

BFP was identified as the primary obesity indicator for autonomic function assessment in Asian young college students, while traditional indices (BMI, WC) provide valuable incremental information. Integrating body composition measurement into youth cardiovascular risk screening is recommended.

## 1. Introduction

Autonomic dysfunction is an early marker of cardiovascular disease (CVD), and the prognostic value of its quantification through heart rate variability (HRV) has been validated in numerous systematic reviews and prospective studies [1, 2]. Lower HRV not only significantly predicts the risk of all‐cause mortality and cardiovascular events [3, 4] but its close association with systemic inflammation and metabolic disturbances also suggests underlying pathophysiological mechanisms [5–7]. Obesity, as a global health challenge, is significantly associated with autonomic dysfunction [8]; however, the complexity of this association extends far beyond what can be explained by the traditional body mass index (BMI) alone [9, 10].

In recent years, novel obesity indices such as the waist‐to‐height ratio (WHtR), body fat percentage (BFP), a body shape index (ABSI), and weight‐adjusted waist index (WWI) have been proposed to more accurately characterize the metabolic risk of obesity from the perspectives of fat distribution and body composition [11, 12]. Consensus documents strongly recommend indicators like waist circumference (WC) as an essential clinical metric [13], while systematic reviews indicate significant limitations of BMI in identifying individuals with excess adiposity (sensitivity approximately 50%) [14]. Furthermore, Asian populations exhibit a distinct obesity phenotype characterized by higher BFP and a stronger tendency toward central obesity at lower BMI levels [15], suggesting that obesity–HRV association models developed primarily in Western populations may not be directly applicable to this group [16]. Particularly during young adulthood, the autonomic nervous system exhibits high plasticity, making this period a potential critical window for early intervention regarding the associations of obesity on autonomic function [17, 18]. However, significant gaps remain in the current research: first, most studies focus on middle‐aged, elderly, or clinical populations [19–21], leading to a severe lack of evidence specifically concerning healthy young adults, especially those of Asian descent; second, the high collinearity among various obesity indices makes it difficult for traditional single‐model analyses to disentangle their independent contributions to HRV; third, although sex differences in HRV are well documented [22, 23], whether the association between obesity and HRV is modified by sex remains unclear in young Asian populations; finally, it is not yet established whether novel body composition/body shape indices provide significant incremental value over traditional indices (BMI, WC) in the assessment of obesity‐related health risks.

This study therefore employed a multimodel validation framework in a large sample of Asian young college students to systematically evaluate the associations of traditional indices (BMI, WC, waist‐to‐hip ratio [WHR]) and novel indices (WHtR, BFP, ABSI, body adiposity index [BAI]) with autonomic function and to address the following scientific questions: (1) What are the associations between various obesity indices and HRV in an Asian young adult population? (2) After controlling for multicollinearity, how are the relative importance of these indices ranked? (3) Does the association between obesity and autonomic function differ by sex? (4) Do novel body composition/body shape indices provide incremental predictive value beyond traditional indices (BMI, WC)? Our findings aim to inform cardiovascular autonomic risk assessment strategies in Asian youth and may guide the selection of appropriate indices for cardiovascular risk screening.

## 2. Methods

### 2.1. Study Population and Sampling

This cross‐sectional observational study was conducted on the campus of Anqing Medical College in China from February 20 to June 10, 2025. We employed a mixed sampling strategy: cluster sampling was used to enroll students from classes requiring a compulsory health science course (*n* = 2298), combined with campus‐wide voluntary recruitment (*n* = 1157). A total of 3455 students were initially recruited. After excluding 124 individuals who did not meet the inclusion criteria, 36 with missing HRV data, and 115 with missing exercise data, 3180 participants (784 males, 2396 females, mean age 19.56 ± 1.02 years, Figure S1) were included in the final analysis. Exclusion criteria included a history of cardiovascular or cerebrovascular diseases, pregnancy, psychiatric disorders, taking medications that might affect the accuracy of the measurements, inability to cooperate with the measurement procedures, and unwillingness to provide written informed consent. The study adhered to the principles of the Declaration of Helsinki and was approved by the Ethics Committee of Anqing Medical College (Approval No. 2025‐02‐004). All participants provided written informed consent, and data were deidentified to protect privacy.

### 2.2. Variable Definition, Measurement, and Quality Control

Weight (Wt, kg), height (Ht, cm), and fat mass (FM, kg, measured by bioelectrical impedance analysis [BIA]) were assessed using an intelligent ultrasonic height–weight measuring instrument (Yolanda CP30B, Shenzhen, China). Waist circumference (WC, cm) and hip circumference (HC, cm) were measured using a standard tape measure. The following obesity indices were calculated based on the above measurements [24–27]: BMI (kg/m^2^) = Wt/Ht^2^; WHR = WC/HC; WHtR = WC/Ht; ABSI (m^1/2^) = WC/(BMI^2/3^ × Ht^1/2^); WWI (cm/kg^1/2^) = WC/Wt^1/2^; BAI (%) =  (HC/Ht^3/2^)−18; BFP(%) =  (FM/Wt) × 100. Unit conversions were applied prior to calculation as required by specific formulas: WC was converted to meters for ABSI and WHtR calculations to ensure consistent units and avoid geometric distortion; for BAI, HC was in cm and Ht was in m. ABSI values are presented as × 10^−3^ m^1/2^ for clarity in statistical presentation as raw values are typically < 0.1.

A conventional 12‐lead ECG device (CONTE 8000G, sampling rate 1000 Hz, Qinhuangdao, China) was used to acquire electrocardiographic indices. A 5 min resting ECG was recorded in the supine position (paper speed: 25 mm/s, calibration: 10 mm/mV). We extracted the following parameters using the device’s proprietary software (version V3.4.7, Wuhan, China): heart rate (HR, bpm), standard deviation of NN intervals (SDNN, ms), root‐mean‐square of successive differences (rMSSD, ms), percentage of NN50 (pNN50, %), standard deviation of successive differences (SDSD, ms), total power (TP, ms^2^), very low frequency power (VLF, ms^2^), low‐frequency power (LF, ms^2^), high‐frequency power (HF, ms^2^), and the LF/HF ratio. Normalized low‐frequency power (LFnorm = 100 × LF/[TP − VLF], nU) and normalized high‐frequency power (HFnorm = 100 × HF/[TP − VLF], nU) were also calculated [2].

The time of ECG data acquisition was recorded and categorized as: morning (8:00 ≤ time < 12:00), afternoon (12:00 ≤ time < 18:00), or evening (18:00 ≤ time < 22:00). Exercise data (total data for the 3 months preceding the ECG assessment) were collected via the smartphone application “Budaolepao” (Version 4.1.3, Leping Sports, Wuhan, China). This app utilizes the phone’s built‐in GPS and accelerometer to record users’ walking/running activities, with data automatically uploaded to the cloud and exported. Total valid exercise distance (TVED, km) was defined by the app’s default algorithm: each single session required a minimum distance of 1.50 km, a pace between 5′00″ and 12′00″ per kilometer, and passing through 2 designated checkpoints. Sessions ≤ 3 km had to be completed within 1 h, and sessions > 3 km but < 10 km within 2 h (including pause time); sessions exceeding these time limits were excluded. The total time spent completing all valid exercise distances constituted the total effective exercise duration (TEED, hours). Average pace (PACE, min/km) was calculated as PACE = (TEED × 60)/TVED [28, 29]. Exercise data, HR, measurement time, and age were included as covariates in the analyses.

All measurements were performed by trained and qualified personnel. Measurements took place indoors in a quiet environment with controlled temperature (22°C–24°C). Participants rested quietly in a seated position for at least 10 min prior to measurement (no strenuous exercise, food intake, or stimulant consumption within the preceding 4 h). The ECG device underwent routine initialization and self‐check. Skin was cleaned with alcohol swabs, and electrodes were placed according to standard 12‐lead ECG positions. Baseline calibration was performed (amplitude > 0.5 mV, noise level < −60 dB, ≥ 95% analyzable normal‐to‐normal intervals, and ΔHR < 10 bpm). If participants questioned their height or weight results, the measurement was repeated and the average value was recorded. The equipment was calibrated according to the manufacturer’s instructions after every 50 participants. Data quality was reviewed every 2 weeks: 10% of the original data were randomly selected, and two physicians independently calculated derived measures. For ECG data, HR was verified, and RR intervals were manually calculated (selecting 3 consecutive sinus rhythm beats from each ECG trace, preferably lead II if clear, excluding beats with arrhythmias like premature contractions or atrial fibrillation). A difference of ≤ 5% between the two physicians′ calculations was considered acceptable [30, 31]; otherwise, the data were remeasured, recorded, and rechecked before inclusion in the analysis.

### 2.3. Statistical Analysis

All statistical analyses were conducted using Python (version 3.12), leveraging libraries including pandas, scipy, statsmodels, scikit‐learn, and seaborn [32]. Normality of variables was assessed using the Shapiro–Wilk test. Based on the results, the distribution of variables was described: continuous variables are presented as mean (SD) or median (IQR), and categorical variables as frequencies (%). Group differences between sexes for continuous variables were examined using the Mann–Whitney *U* test, with Cliff’s delta (*δ*) calculated as the effect size (|*δ*| ≈ 0.15, 0.33, and 0.47 indicating small, medium, and large effects, respectively) [33]. Spearman’s rank or Pearson correlation analyses were conducted, and sex‐stratified heatmaps were generated for obesity indicators, HRV indicators, and their intercorrelations. Given the correlational structure among HRV indicators, the Kaiser–Meyer–Olkin (KMO) test was employed to assess the suitability for dimensionality reduction [34]. Principal component analysis (PCA) was subsequently performed on the entire sample and on sex‐stratified subsamples to identify the core dimensions of the HRV indicators. Principal components were extracted based on Kaiser’s criterion (eigenvalue > 1), considering factor loading strength (|loading| > 0.4), stability across sexes, and clinical relevance, to select representative indicators for each dimension as dependent variables [35]. The Akaike Information Criterion (AIC) was used to determine the single optimal physical activity covariate for all subsequent models [36]. The primary analysis consisted of three core components: first, sex‐stratified single‐predictor multivariable models [37] were used to initially assess the independent association of each standardized obesity indicator with the HRV indicators, reporting the standardized regression coefficient (*β*), 95% confidence interval (CI) obtained via bootstrapping (1000 resamples), *p* value, and explained variance (*R* ^2^); this step aimed to identify candidate predictors and evaluate their signal strength. Second, a multimodel comparison and performance evaluation was implemented [38]. To address potential multicollinearity, a four‐track parallel model comparison strategy was adopted, including (1) best subset regression: comparing Δ*R* ^2^ and ΔAIC of parsimonious models representing different physiological dimensions to assess the overall predictive performance [39]; (2) random forest permutation importance analysis: quantifying the independent contribution of each indicator to the predictive model using a collinearity‐resistant algorithm [40]; (3) elastic net regression: optimizing regularization parameters using 5‐fold cross‐validation (testing l1_ratio range 0.1–1.0 and alpha values at 100 logarithmic points from 10^−4^ to 10^1^), determining the optimal penalty structure via grid search, and performing variable selection through cross‐validation to verify the robustness of core indicators [41]; and (4) incremental value test: directly evaluating the incremental predictive value (ΔR^2^) provided by adding each novel indicator to the best single‐indicator model [42]. Finally, tests for effect modification by sex were conducted; only indicators suggesting potential interaction (*p* < 0.10) in the univariable analysis were included in an “indicator × sex” interaction term in models using the full sample, and the false discovery rate (FDR) for multiple testing was controlled using the Benjamini–Hochberg method (FDR *p* < 0.05) [43]. All models were adjusted for basic covariates including age, HR, physical activity behavior, and measurement time. Based on the core obesity indices identified by the four‐track parallel analysis, subgroup analyses were performed using multiple linear regression to compare HRV indices across groups. Receiver operating characteristic (ROC) curves were generated, and the area under the curve (AUC) was calculated with 95% confidence intervals estimated using the bootstrap method (1000 resamples) to assess how well obesity indices could distinguish low HRV risk. Extensive sensitivity analyses will be performed to assess the robustness of the results, including substituting the HRV outcome measures, replacing the physical activity covariate, and repeating the analyses after excluding extreme BMI values.

## 3. Results

### 3.1. Descriptive Statistics and Variable Selection

As shown in Table 1, this study included a total of 3180 participants (784 males, 2396 females). Widespread sex differences were observed in body morphology and composition indicators: males had significantly higher values for Ht, Wt, WC, WHR, and ABSI (*δ* = 0.868, 0.591, 0.563, 0.643, 0.499, respectively), while females had higher BFP and BAI (*δ* = −0.487, −0.428, respectively). Regarding exercise performance, males had a higher TVED and a faster PACE (*δ* = 0.390, −0.447, respectively), but no significant sex difference was found for TEED. For basic physiological indicators, resting HR showed a significant but minimal sex difference (*δ* = −0.051). HRV analysis indicated that males had higher LF, LF/HF ratio, and TP (*δ* = 0.170, 0.365, 0.054, respectively), whereas females had higher HF, rMSSD, and pNN50 (*δ* = −0.115, −0.074, −0.082, respectively). Additionally, the sex difference in FM was significant but very small in effect size (*δ* = −0.054).

**TABLE 1:** Baseline characteristics by sex.

Variable	Median (Q1, Q3)			*p*	FDR *p*	*δ* (95% CI)
	Overall (*N* = 3180)	Male (*n* = 784)	Female (*n* = 2396)			
Ht (cm)	163.00 (159.00, 169.50)	174.00 (169.50, 177.50)	161.50 (157.50, 165.00)	< 0.001	< 0.001	0.868 (0.849, 0.886)
Wt (kg)	58.80 (52.20, 68.70)	70.50 (61.38, 80.72)	56.10 (50.60, 63.52)	< 0.001	< 0.001	0.591 (0.557, 0.625)
FM (kg)	14.75 (11.50, 19.60)	14.65 (9.60, 20.70)	14.80 (11.80, 19.10)	0.024	0.028	−0.054 (−0.110, −0.003)
HC (cm)	94.00 (89.30, 100.00)	97.00 (91.20, 103.12)	93.00 (88.90, 98.50)	< 0.001	< 0.001	0.261 (0.215, 0.304)
BMI (kg/m^2^)	21.89 (19.79, 25.00)	23.25 (20.49, 26.79)	21.57 (19.60, 24.37)	< 0.001	< 0.001	0.223 (0.176, 0.265)
WC (cm)	71.45 (66.00, 80.00)	80.80 (73.00, 90.00)	69.80 (64.60, 76.00)	< 0.001	< 0.001	0.563 (0.528, 0.601)
WHR (−)	0.77 (0.73, 0.82)	0.83 (0.79, 0.89)	0.75 (0.71, 0.79)	< 0.001	< 0.001	0.643 (0.610, 0.676)
BFP (%)	25.51 (21.47, 29.68)	20.69 (15.26, 25.94)	26.46 (23.00, 30.51)	< 0.001	< 0.001	−0.487 (−0.529, −0.445)
WHtR (−)	0.44 (0.40, 0.48)	0.46 (0.42, 0.52)	0.43 (0.40, 0.47)	< 0.001	< 0.001	0.296 (0.250, 0.343)
ABSI (m^1/2^)	71.58 (68.25, 75.00)	75.13 (72.00, 78.19)	70.61 (67.49, 73.47)	< 0.001	< 0.001	0.499 (0.460, 0.536)
BAI (%)	26.83 (24.18, 29.73)	24.25 (21.93, 27.42)	27.46 (25.13, 30.24)	< 0.001	< 0.001	−0.428 (−0.468, −0.385)
WWI (cm/kg^1/2^)	9.36 (8.93, 9.84)	9.64 (9.17, 10.17)	9.28 (8.87, 9.74)	< 0.001	< 0.001	0.286 (0.243, 0.333)
TVED (km)	119.42 (109.67, 142.25)	147.74 (117.23, 182.50)	114.84 (109.56, 138.09)	< 0.001	< 0.001	0.390 (0.340, 0.440)
TEED (h)	20.65 (17.64, 24.52)	22.06 (17.26, 27.55)	20.49 (17.78, 23.94)	< 0.001	< 0.001	0.139 (0.084, 0.190)
PACE (min/km)	10.45 (9.47, 10.96)	9.67 (8.24, 10.45)	10.62 (9.81, 11.07)	< 0.001	< 0.001	−0.447 (−0.484, −0.406)
HR (bpm)	75.00 (68.00, 82.25)	74.00 (66.00, 83.00)	75.00 (68.00, 82.00)	0.032	0.035	−0.051 (−0.099, −0.002)
SDNN (ms)	49.22 (37.70, 63.83)	50.78 (38.28, 67.25)	48.84 (37.52, 62.82)	0.033	0.036	0.051 (0.006, 0.098)
rMSSD (ms)	40.74 (26.90, 59.90)	38.20 (24.17, 56.92)	41.45 (27.71, 60.41)	0.002	0.002	−0.074 (−0.115, −0.023)
SDSD (ms)	49.19 (37.66, 63.97)	50.76 (38.24, 67.23)	48.78 (37.42, 62.74)	0.034	0.036	0.050 (−0.001, 0.092)
TP (ms^2^)	1142.53 (658.44, 1937.65)	1229.32 (671.79, 2149.31)	1119.62 (651.59, 1864.12)	0.023	0.028	0.054 (0.007, 0.103)
LF (ms^2^)	265.35 (152.19, 487.57)	326.81 (187.41, 577.35)	251.00 (143.62, 454.83)	< 0.001	< 0.001	0.170 (0.126, 0.217)
HF (ms^2^)	317.19 (133.65, 665.55)	254.63 (105.16, 573.44)	333.33 (146.11, 690.14)	< 0.001	< 0.001	−0.115 (−0.160, −0.068)
LF/HF (−)	0.86 (0.50, 1.54)	1.34 (0.78, 2.34)	0.76 (0.45, 1.29)	< 0.001	< 0.001	0.365 (0.320, 0.406)
pNN50 (%)	8.51 (2.22, 18.33)	7.32 (1.75, 16.34)	8.93 (2.43, 19.42)	< 0.001	< 0.001	−0.082 (−0.127, −0.037)
LFnorm (nU)	38.33 (27.53, 50.57)	46.01 (34.68, 57.07)	36.26 (26.05, 47.85)	< 0.001	< 0.001	0.295 (0.250, 0.338)
HFnorm (nU)	45.22 (30.43, 58.77)	35.48 (22.72, 49.09)	48.40 (33.99, 61.00)	< 0.001	< 0.001	−0.325 (−0.370, −0.283)

*Note:* Data are presented as median (IQR). Group comparisons were performed using the Mann–Whitney *U* test. Cliff’s delta (*δ*) was calculated as a nonparametric measure of effect size, with 95% CI estimated via bootstrap method (1000 resamples). The magnitude of the effect size was interpreted as follows: |*δ*| ≈ 0.15 (small), 0.33 (medium), and 0.47 (large). The FDR procedure was applied to adjust *p* values for multiple comparisons. Both raw *p* values and FDR‐adjusted *p* values (FDR *p*) are presented. Statistical significance was defined as FDR *p* < 0.05. For clarity of presentation, ABSI values are presented as × 10^−3^ m^1^⁄^2^ for clarity.

**FIGURE 1:**
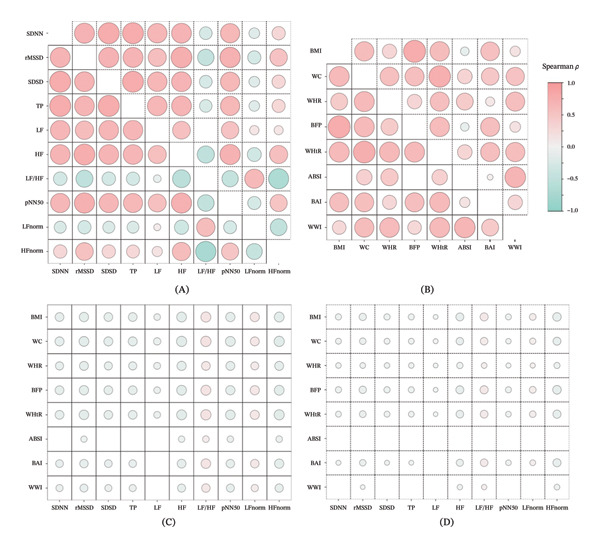
Sex‐stratified heatmaps of correlation matrices between indicators (*N* = 3180). The figure displays only indicator pairs with statistically significant correlations (FDR *p* < 0.05). Bubble size represents the absolute value of the correlation coefficient, and color indicates the direction of correlation (teal: negative correlation; pink: positive correlation). Solid borders denote male participants, while dashed borders denote female participants. (A) Correlation matrix among HRV parameters (lower‐left triangle: males; upper‐right triangle: females). (B) Correlation matrix among obesity indicators (lower‐left triangle: males; upper‐right triangle: females). (C) Correlation matrix between obesity indicators and HRV parameters in males. (D) Correlation matrix between obesity indicators and HRV parameters in females. All nonsignificant correlation pairs and same‐indicator pairs (diagonal cells in A and B) are left blank. Complete correlation coefficients and statistical test results are provided in supporting Table S1.

Correlation analysis (Figure 1, Table S1) suggested systematic correlation patterns between obesity indicators and HRV parameters among the 3180 Asian young college students, with evident sex differences. In the male subsample, all obesity indicators except ABSI (i.e., BMI, WC, WHR, BFP, WHtR, BAI, WWI) showed negative correlations with most HRV parameters (*ρ* = −0.1070 to −0.2175) but positive correlations with the LF/HF ratio (*ρ* = 0.1513–0.2237). ABSI showed only weak correlations with rMSSD (*ρ* = −0.0837), HF (*ρ* = −0.0859), LF/HF (*ρ* = 0.0932), and pNN50 (*ρ* = −0.0887). In the female subsample, the correlation pattern was similar to males but weaker in magnitude, with the absolute values of correlation coefficients between obesity indicators and HRV parameters mostly below 0.16. ABSI showed no significant correlation with any HRV parameter (|*ρ*| < 0.03). We found significant correlations among the obesity indicators themselves: in males, BMI and BFP were nearly perfectly correlated (*ρ* = 0.9997), and WC and WHtR were highly correlated (*ρ* = 0.9691); in females, BMI and BFP were highly correlated (*ρ* = 0.9938), and WC and WHtR were very highly correlated (*ρ* = 0.9518). All obesity indicators were significantly positively correlated with each other. HRV parameters showed high internal structural correlations: SDNN and SDSD were nearly perfectly correlated in both sexes (male *ρ* = 0.9999, female *ρ* = 0.9999), and HF and rMSSD were strongly correlated (male *ρ* = 0.9831, female *ρ* = 0.9760). LF/HF was negatively correlated with HF (male *ρ* = −0.7167, female *ρ* = −0.6626) and positively correlated with LFnorm (male *ρ* = 0.7599, female *ρ* = 0.8236).

PCA performed on the 10 HRV indicators (Table 2) indicated that the data were suitable for factor analysis (overall KMO = 0.78). Despite computational anomalies in Bartlett’s test of sphericity due to high mathematical correlations among HRV indicators (e.g., near‐perfect collinearity between SDNN and SDSD, *r* = 0.9999), three principal components were extracted based on the eigenvalue > 1 criterion (Kaiser’s criterion), cumulatively explaining 87.35% of the total variance. This structure was highly consistent across male and female subgroups (variance explained: 87.44% and 87.65%, respectively). PC1 (62.23%) loaded highest on time‐domain indicators (e.g., SDNN, rMSSD), representing “General Autonomic Function”; PC2 (19.62%) was dominated by the LF/HF ratio and normalized powers (LFnorm, HFnorm), reflecting “Frequency‐domain Balance”; PC3 (5.50%) was primarily loaded by LFnorm, representing “Spectral Domain Characteristics” independent of the overall balance. Based on factor loading strength, cross‐sex stability, and clinical consensus, SDNN, LF/HF, and LFnorm were selected as representative indicators for these three dimensions, respectively, for subsequent analysis.

**TABLE 2:** PCA of HRV indices: factor loading matrix.

HRV index	Overall (*N* = 3180)			Male (*n* = 784)			Female (*n* = 2396)		
	PC1	PC2	PC3	PC1	PC2	PC3	PC1	PC2	PC3
SDNN (ms)	0.383	0.148	0.019	0.378	0.155	0.066	0.383	0.142	−0.015
rMSSD (ms)	0.382	0.007	0.106	0.372	−0.001	0.186	0.383	0.014	−0.064
SDSD (ms)	0.383	0.148	0.019	0.378	0.155	0.066	0.383	0.142	−0.015
TP (ms^2^)	0.37	0.206	0.106	0.363	0.23	−0.011	0.37	0.2	−0.182
LF (ms^2^)	0.287	0.37	−0.401	0.272	0.413	−0.444	0.297	0.355	0.341
HF (ms^2^)	0.351	0.072	0.323	0.355	0.056	0.218	0.348	0.087	−0.364
LF/HF	−0.161	0.503	0.604	−0.176	0.494	0.706	−0.161	0.514	−0.44
pNN50 (%)	0.329	−0.133	−0.163	0.337	−0.106	−0.02	0.325	−0.135	0.315
LFnorm (nU)	−0.161	0.523	−0.552	−0.178	0.519	−0.453	−0.156	0.526	0.613
HFnorm (nU)	0.242	−0.477	−0.125	0.262	−0.448	−0.083	0.24	−0.477	0.213
Variance Explained (%)	62.23	19.62	5.5	63.6	17.93	5.9	62.71	19.45	5.49
Cumulative Variance Explained (%)	62.23	81.85	87.35	63.6	81.53	87.44	62.71	82.16	87.65

*Note:* PCA was performed on 10 HRV indices. Components with eigenvalues greater than 1 were retained, resulting in three principal components (PCs) that collectively explained 87.35% of the total variance. Varimax rotation was applied to achieve a simpler and more interpretable factor structure. Factor loadings with an absolute value greater than 0.4 are considered practically significant. The structure was highly consistent across sex‐specific subgroups (male: 87.44%; female: 87.65% variance explained). PC1 represents general autonomic function (high loadings on time‐domain indices), PC2 represents frequency‐domain balance (high loadings on LF/HF ratio and normalized powers), and PC3 represents spectral domain characteristics (dominant loading on LFnorm).

The predictive power of TVED, TEED, and PACE for the three representative HRV indicators was compared based on the AIC (Table S2). Detailed results showed different advantages for each variable: TVED provided the best fit for SDNN, TEED was best for LF/HF, while PACE showed the greatest advantage for LFnorm (ΔAIC = 4.87 versus the next best model). For a comprehensive assessment, the sum of AIC values across all HRV outcomes was calculated for each variable. PACE had the lowest total AIC (∑AIC = 68,681.8), indicating its superior overall predictive performance. Given that PACE is a direct indicator of exercise intensity, is physiologically more sensitive in reflecting autonomic regulation (particularly sympathetic activity), and demonstrated the best statistical performance, it was ultimately selected as the core physical activity covariate for subsequent analyses to ensure models possessed both physiological interpretability and statistical robustness.

### 3.2. Single‐Predictor Multivariable Model Analysis

**FIGURE 2:**
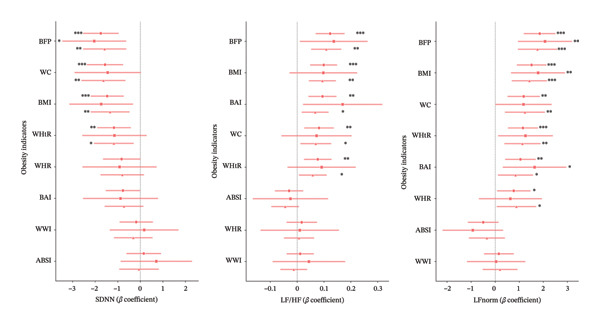
Association between standardized obesity indicators and HRV parameters across three autonomic function (*N* = 3180) domains. Tornado plots display standardized β coefficients (points) and 95% CIs (horizontal lines) from sex‐stratified linear regression models adjusted for age, HR, physical activity (PACE), and measurement time. Data points represent results from different analytical groupings: circles = total sample, squares = male subgroup, triangles = female subgroup. Obesity indicators are sorted in descending order of absolute β coefficient magnitude from the total sample analysis (strongest association at the top). Significance levels: ^∗^FDR *p* < 0.05, ^∗∗^FDR *p* < 0.01, ^∗∗∗^FDR *p* < 0.001 after false discovery rate correction.

Standardized obesity indicators showed significant and comparable associations with HRV parameters (Table 3, Figure 2, Table S3). General autonomic function (SDNN) was negatively associated with most obesity indicators, with BFP showing the strongest association (*β* = −1.76, 95% CI: −2.55 to −0.97, FDR *p* < 0.001), followed by WC (*β* = −1.57) and BMI (*β* = −1.47). LF/HF was positively associated with BAI, BMI, WC, and WHtR (*β* = 0.058, 0.123, FDR *p* < 0.05). LFnorm was positively associated with BFP, BMI, WC, WHtR, and BAI (*β* = 1.04, 1.84, FDR *p* < 0.05), with BFP having the most prominent effect (LF/HF: *β* = 0.123; LFnorm: *β* = 1.84). Sex‐stratified analysis indicated that the effect sizes for the associations of BFP with SDNN (*β* = −2.05) and LFnorm (*β* = 2.06) were stronger in males, while associations between central obesity indicators like WC and WHtR and HRV were involved in a broader range of indicators in females. ABSI and WWI did not reach significance in any model (FDR *p* > 0.05).

**TABLE 3:** Sex‐stratified associations of standardized obesity indicators with HRV parameters.

HRV parameter	Sex	Obesity indicator	*N*	*β* (95% CI)	FDR *p*	*R* ^2^
SDNN (ms)	Overall	BFP	3180	−1.76 (−2.55, −0.97)	< 0.001	0.278
		WC	3180	−1.57 (−2.38, −0.77)	0.0004	0.277
		BMI	3180	−1.47 (−2.21, −0.74)	< 0.001	0.277
		WHtR	3180	−1.17 (−1.92, −0.42)	0.004	0.275
	Male	BFP	784	−2.05 (−3.46, −0.63)	0.038	0.266
	Female	BFP	2396	−1.59 (−2.55, −0.62)	0.005	0.282
		WC	2396	−1.64 (−2.62, −0.66)	0.005	0.282
		BMI	2396	−1.34 (−2.20, −0.48)	0.006	0.282
		WHtR	2396	−1.18 (−2.06, −0.29)	0.018	0.281

LF/HF	Overall	BFP	3180	0.123 (0.069, 0.177)	< 0.001	0.231
		BAI	3180	0.094 (0.042, 0.146)	0.001	0.229
		BMI	3180	0.099 (0.048, 0.149)	< 0.001	0.23
		WC	3180	0.081 (0.026, 0.137)	0.006	0.228
		WHtR	3180	0.077 (0.025, 0.128)	0.006	0.228
	Female	BFP	2396	0.108 (0.052, 0.164)	0.001	0.202
		BMI	2396	0.094 (0.043, 0.144)	0.001	0.202
		WC	2396	0.069 (0.012, 0.127)	0.035	0.199
		BAI	2396	0.067 (0.017, 0.117)	0.024	0.2
		WHtR	2396	0.058 (0.006, 0.110)	0.044	0.199

LFnorm (nU)	Overall	BFP	3180	1.84 (1.18, 2.50)	< 0.001	0.135
		BMI	3180	1.51 (0.89, 2.12)	< 0.001	0.133
		WC	3180	1.18 (0.50, 1.85)	0.001	0.13
		WHtR	3180	1.15 (0.52, 1.77)	< 0.001	0.13
		BAI	3180	1.04 (0.41, 1.68)	0.002	0.129
		WHR	3180	0.77 (0.07, 1.46)	0.04	0.128
	Male	BFP	784	2.06 (0.94, 3.19)	0.003	0.099
		BMI	784	1.78 (0.65, 2.90)	0.008	0.095
		BAI	784	1.63 (0.31, 2.95)	0.042	0.09
	Female	BFP	2396	1.75 (0.94, 2.57)	< 0.001	0.108
		BMI	2396	1.41 (0.68, 2.15)	< 0.001	0.107
		WC	2396	1.23 (0.40, 2.06)	0.008	0.105
		WHtR	2396	1.13 (0.37, 1.88)	0.008	0.105
		BAI	2396	0.84 (0.11, 1.57)	0.038	0.103
		WHR	2396	0.88 (0.06, 1.70)	0.047	0.103

*Note:* Results are derived from sex‐stratified linear regression models. Each model included one standardized obesity indicator as the predictor, adjusted for age, HR, physical activity (PACE), and measurement time. *β*, standardized regression coefficient; CI, confidence interval; FDR *p*, *p* value adjusted for multiple testing across eight obesity indicators using the Benjamini–Hochberg procedure; *R* ^2^, coefficient of determination for the model.

Based on the association strength hierarchy from univariate analyses (BFP > WC ≈ BMI > WHtR > BAI), we further implemented a four‐track modeling strategy: (1) best‐subset regression compared models such as “BFP alone” versus “BFP + WHtR” using ΔR^2^ and ΔAIC to assess the incremental value of WHtR; (2) Random forest with permutation importance was used to quantify the independent contribution of each obesity index to HRV variance, aiming to validate the dominant role of BFP; (3) elastic net regression performed variable selection to test the robustness of BFP and WHtR; and (4) incremental value was directly evaluated by adding each index to the best‐performing univariate model. Additionally, we tested interactions between sex and indices showing suggestive associations in univariate models (*p* < 0.10), with *p* values adjusted for FDR.

### 3.3. Four‐Track Parallel Analysis

**FIGURE 3:**
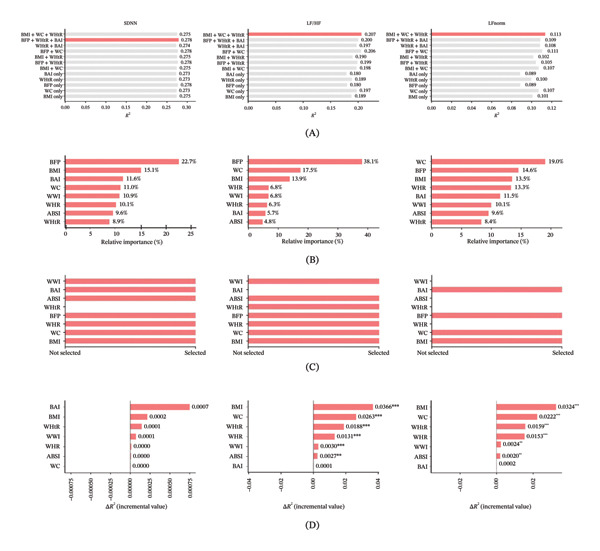
Four‐track parallel analysis evaluating obesity indicators for predicting HRV (*N* = 3180). (A) Best subset regression showing *R* ^2^ values for different predictor combinations. (B) Relative importance analysis quantifying the percentage contribution of each indicator. (C) Elastic net variable selection with cross‐validation. (D) Incremental predictive value of adding each indicator to a baseline model. Highlighted elements indicate optimal or selected components. All analyses are based on total sample and adjusted for covariates. ^∗^FDR *p* < 0.05, ^∗∗^FDR *p* < 0.01, ^∗∗∗^FDR *p* < 0.001. All models were adjusted for age, HR, physical activity (PACE), and measurement time.

Best subset regression identified BFP, WHtR, and BAI combined as the optimal model for predicting SDNN (*R* ^2^ = 27.83%, Figure 3; Table S4). For LF/HF and LFnorm, the optimal models comprised BMI, WC, and WHtR, explaining 20.67% and 11.35% of the variance, respectively. Relative importance analysis showed that BFP contributed the most to predicting LF/HF (38.1%), followed by WC (17.5%) and BMI (13.9%). Elastic net regression results indicated that BFP, BMI, and WC were retained (nonzero coefficients) in the final models across all HRV dimensions, with cross‐validated *R* ^2^ values of 28.06% for SDNN, 22.37% for LF/HF, and 11.69% for LFnorm. Incremental value tests showed that adding BMI to a baseline model containing BFP significantly improved the prediction of LF/HF (Δ*R* ^2^ = 0.0366, FDR *p* < 0.001) and LFnorm (Δ*R* ^2^ = 0.0324, FDR *p* < 0.001) but provided limited improvement for SDNN. The incremental value of WHtR and BAI was weaker. Analysis of sex interaction did not reveal any significant interaction between the obesity indicators and sex (all FDR *p* > 0.05).

### 3.4. Subgroup Analysis

Based on the core obesity indices identified by the four‐track parallel analysis (BFP, BMI, WC) and the criteria of the 2024 Chinese Guidelines for the Diagnosis and Treatment of Obesity [44], participants were divided into four groups using BMI and BFP: normal weight‐normal BFP (reference group, BMI < 24 kg/m^2^ and BFP below sex‐specific thresholds), normal weight obesity (NWO, BMI < 24 kg/m^2^ but BFP ≥ sex‐specific thresholds), overweight/obese‐normal BFP (BMI ≥ 24 kg/m^2^ but BFP < thresholds), and overweight/obese‐elevated BFP (BMI ≥ 24 kg/m^2^ and BFP ≥ thresholds). BFP thresholds were 25% for males and 30% for females. After adjusting for age, HR, PACE, measurement time, and sex using multiple linear regression, adjusted means and group comparisons are shown in Table 4.

**TABLE 4:** HRV indices across BMI and BFP subgroups.

Group	*n*	SDNN (ms)	Difference (95% CI)	FDR *p*	LF/HF	Difference (95% CI)	FDR *p*	LFnorm (nU)	Difference (95% CI)	FDR *p*
Reference	2148	53.67	—	—	1.01	—	—	36.50	—	—
NWO	26	51.70	−1.97 (−10.02, 6.08)	0.631	1.40	0.39 (−0.16, 0.94)	0.210	39.71	3.21 (−3.50, 9.93)	0.391
Overweight/Obese‐normal BFP	139	49.26	−4.41 (−8.13, −0.68)	0.046	1.23	0.22 (−0.03, 0.48)	0.158	38.73	2.23 (−0.87, 5.34)	0.210
Overweight/Obese‐elevated BFP	867	51.39	−2.28 (−3.93, −0.64)	0.020	1.19	0.18 (0.07, 0.29)	0.008	39.92	3.42 (2.05, 4.79)	< 0.001

*Note:* *p* values were adjusted for FDR using the Benjamini–Hochberg procedure across nine comparisons (3 groups × 3 HRV indices). Difference from reference group, adjusted for age, HR, physical activity (PACE), measurement time, and sex. Reference group: normal weight with normal BFP (BMI < 24 kg/m^2^ and BFP below sex‐specific thresholds). NWO: normal weight obesity (BMI < 24 kg/m^2^ but BFP ≥ sex‐specific thresholds). It should be noted that the NWO subgroup has a small sample size (*n* = 26), which limits the statistical power to detect significant differences compared to the reference group. BFP thresholds: 25% for males, 30% for females.

Compared with the reference group, the overweight/obese‐elevated BFP group showed significant differences in all three HRV indices after FDR correction for multiple comparisons (SDNN: adjusted mean difference −2.28 ms, 95% CI: −3.93 to −0.64, FDR *p* = 0.020; LF/HF: adjusted mean difference 0.18, 95% CI: 0.07 to 0.29, FDR *p* = 0.008; LFnorm: adjusted mean difference 3.42, 95% CI: 2.05–4.79, FDR *p* < 0.001). The overweight/obese‐normal BFP group showed a significant reduction only in SDNN (adjusted mean difference −4.41 ms, 95% CI: −8.13 to −0.68, FDR *p* = 0.046). The NWO group showed trends in the expected direction (lower SDNN, higher LF/HF, and LFnorm) but did not reach statistical significance after FDR correction (all FDR *p* > 0.05), possibly due to the small sample size (*n* = 26).

### 3.5. ROC Analysis

To explore clinical reference thresholds for identifying low HRV risk using core obesity indices, low HRV risk was defined as the lowest 20th percentile of SDNN distribution (35.18 ms, *n* = 636). ROC curves were generated, and 95% CIs for AUC were estimated using the bootstrap method (1000 resamples). As shown in Table 5, BFP, BMI, and WC all showed low discriminative ability for low HRV risk (AUC range 0.54–0.57). BFP had a slightly higher AUC than BMI and WC (0.571 vs. 0.563 vs. 0.540). Based on the Youden index, the optimal cutoff for BFP was 27.3%, with a sensitivity of 46.9% and a specificity of 64.8%. Using SDNN < 50 ms as an alternative definition gave similar results (BFP AUC = 0.541, 95% CI: 0.521–0.562, cutoff = 24.6%), though AUC values were lower.

**TABLE 5:** ROC analysis of core obesity indices for identifying low HRV risk.

Obesity index	AUC (95% CI)	Optimal cutoff	Sensitivity (%)	Specificity (%)	Youden index
*Low HRV risk defined as lowest 20th percentile of SDNN (n = 636)*					
BFP	0.571 (0.546–0.596)	27.3%	46.9	64.8	0.117
BMI	0.563 (0.538–0.588)	24.3 kg/m^2^	38.7	73.0	0.116
WC	0.540 (0.513–0.566)	84.0 cm	23.7	84.1	0.079

*Low HRV risk defined as SDNN < 50 ms (n = 1645)*					
BFP	0.541 (0.521–0.562)	24.6%	60.2	48.6	0.088
BMI	0.531 (0.511–0.551)	20.4 kg/m^2^	70.1	35.5	0.056
WC	0.522 (0.502–0.542)	83.3 cm	20.4	84.1	0.045

*Note:* 95% CIs for AUC were estimated using the bootstrap method (1000 resamples).

### 3.6. Sensitivity Analysis

The results of the sensitivity analysis (Table S5) confirmed the high robustness of the main findings. When substituting HRV indicators (using rMSSD, HFnorm, pNN50 instead of the original three dimensions), excluding extreme BMI values (outside 16–35 kg/m^2^, excluding 65 cases, remaining *n* = 3115), using TVED instead of PACE as the physical activity covariate, and in combined sensitivity scenarios, the core obesity indicators BFP, BMI, and WC consistently maintained important predictive status. Specifically, BFP was retained as a key predictor in 8 out of 12 sensitivity analysis scenarios, WC was retained in 11/12 scenarios, and BMI was retained in 9/12 scenarios; model predictive performance remained stable (cross‐validated *R* ^2^ ranges: SDNN/rMSSD 25.9%–26.5%, LF/HF/HFnorm 21.6%–35.1%, LFnorm/pNN50 3.9%–46.3%); and the variable selection patterns were highly consistent across different sensitivity scenarios, indicating that the main results were not influenced by the choice of HRV indicators, exclusion of extreme values, or measurement of the physical activity covariate.

## 4. Discussion

In this cross‐sectional study of 3180 Asian young college students (75.3% female), we used PCA to reduce HRV indicators to three dimensions: general function (SDNN), frequency‐domain balance (LF/HF), and frequency‐domain characteristics (LFnorm). A multimodel framework was used to assess their associations with obesity indices. BFP showed negative associations with SDNN and positive associations with LF/HF, with the strongest associations. Relative importance analysis showed that BFP contributed most to LF/HF (38.1%), higher than WC (17.5%) and BMI (13.9%). Sex‐stratified analysis suggested stronger associations for BFP in males and for WC/WHtR in females, but interaction tests were not significant (FDR *p* > 0.05). Incremental value tests showed that adding BMI to BFP improved prediction for LF/HF and LFnorm, while ABSI and WWI showed no robust independent value. Together, these findings support cardiovascular autonomic risk assessment in Asian youth and suggest prioritizing BFP with WC and BMI as complements.

Our findings align with previous reports and extend them to Asian youth. Multiple studies suggest that indicators directly reflecting fat content or distribution are better than BMI for predicting autonomic function. Poliakova et al. [45] found that DXA‐measured BFP was the obesity index most strongly correlated with HRV in abdominally obese men. Habib et al. [46] also confirmed that visceral fat rating had a stronger negative correlation with HRV than BMI. Similar observations have been reported in Asian populations from India, Korea, and Saudi Arabia [47–49]. Our study strengthens this conclusion in Asian youth: BFP is the core obesity indicator for predicting HRV. Multimodel analysis further showed that although BFP contributed the most (e.g., 38.1% for LF/HF) and was consistently retained across models, WC and BMI still provided independent information. Model differences reflected the complementary nature of these indicators. For SDNN, the best subset regression selected BFP + WHtR + BAI, suggesting that WHtR and BAI can supplement BFP in a parsimonious model. For LF/HF and LFnorm, although best subset regression selected BMI, WC, and WHtR, relative importance analysis and elastic net consistently showed that BFP had the largest contribution. Incremental value tests showed that adding BMI to BFP significantly improved prediction accuracy for LF/HF and LFnorm (Δ*R* ^2^ > 0.03, FDR *p* < 0.001). Together, BFP is the most robust core indicator for predicting HRV; WC (reflecting central obesity) and BMI (possibly reflecting lean mass or mechanical load) are important supplements, especially for assessing LF/HF. Subgroup analysis showed that overweight/obese individuals with elevated BFP had significantly impaired HRV across all indices, while those with normal BFP showed reduced SDNN only, suggesting that elevated body fat is a key factor in HRV impairment independent of BMI. BAI may be limited to SDNN, and WHtR also contributes to some extent. These findings are consistent with Gómez‐Ambrosi et al. [50]: the unique obesity phenotype of Asian populations [15, 16] makes BFP more sensitive for capturing early metabolic risk. ABSI and WWI showed no independent associations across HRV dimensions, suggesting limited predictive value in this population.

The central role of BFP supports the “obesity–inflammation–autonomic nervous system” hypothesis. Excess adipose tissue, particularly visceral fat, triggers low‐grade inflammation through free fatty acids and proinflammatory cytokines [5, 6]. This inflammatory state has been linked to reduced HRV [51, 52] and may interfere with autonomic function [53]. However, we did not measure inflammatory markers, so this hypothesis requires further testing. Regarding sex differences, our interaction models detected no significant “indicator × sex” interaction (FDR *p* > 0.05), suggesting consistent association patterns across sexes. However, stratified analyses suggested potential heterogeneity: males appeared more sensitive to BFP, while females showed broader associations with WC and WHtR. This may relate to biological differences in body composition, hormones, or fat metabolism [54, 55] and requires validation in larger samples. This differs from some Western adult studies that reported significant sex modification [23]. ROC analysis gave a BFP cutoff of 27.3% for identifying low HRV risk, slightly above the 25% threshold recommended by Chinese guidelines, suggesting that existing thresholds may need reconsideration for autonomic risk.

It is important to interpret the ROC findings with caution. The AUC values for BFP, BMI, and WC were modest (range: 0.54–0.57), indicating that these anthropometric indices alone have modest discriminative ability for low HRV at the individual level. However, for population‐based screening purposes, identifying even a modest shift in risk distribution can be valuable for public health interventions. This is not surprising given the multifactorial nature of HRV, which is also regulated by genetics, fitness levels, and lifestyle factors not fully captured in this study. Consequently, these obesity indices should be viewed as risk stratification tools rather than diagnostic tests for autonomic function. The derived cutoff (BFP 27.3%) may serve as a preliminary reference for screening “high‐risk” phenotypes within this young college population, but it lacks the precision required for clinical diagnosis.

Our findings have clear clinical and public health value. They support moving beyond BMI in youth health screening [9, 10] and incorporating body composition measures (e.g., BIA) as routine tools to help identify young individuals with high body fat at risk for autonomic function. Given the high plasticity of the autonomic nervous system during youth [17, 18], early intervention could provide a window for preventing CVD. Public health strategies should consider sex factors cautiously.

## 5. Limitations

This study has several limitations. (1) The cross‐sectional design cannot infer causality, nor can it rule out reverse causation (autonomic dysfunction may affect energy metabolism and eating behaviors). (2) The use of 5‐min short‐term HRV instead of 24‐h monitoring, while convenient for large‐scale application, may not capture circadian rhythms of autonomic function [56]. Moreover, breathing frequency was not controlled, which may affect HF power and the LF/HF ratio; thus, interpretation of frequency‐domain markers should be cautious. (3) Body fat assessed by BIA has inherent measurement error; future studies could use more accurate methods such as DXA [10]. (4) This study did not include inflammatory markers, so the “adipose–inflammation–autonomic nervous system” pathway could not be directly tested [52]. (5) The normal weight obesity (NWO) group had only 26 participants, a small sample size that may have limited statistical power for detecting subgroup differences. (6) The ROC analysis showed modest discriminative accuracy for all obesity indices (AUC range: 0.54–0.57; 95% CIs were wide). This suggests that these anthropometric measures alone have limited ability to identify individuals with low HRV, and the derived cutoff (BFP 27.3%) requires validation in independent samples before clinical application. (7) Some group differences in this study reached statistical significance, but the effect sizes were small (e.g., SDNN differences of 2–4 ms); their clinical relevance requires further investigation using prospective outcome studies. (8) The sample came from a single region, so generalizing the conclusions to other populations requires caution. Future studies could use prospective designs to explore causal relationships between obesity indices and HRV changes and incorporate biomarkers such as inflammatory factors to elucidate underlying mechanisms. Validation across different age groups and ethnic populations would help develop more targeted risk assessment and intervention strategies.

## 6. Conclusions

Using a multimodel validation framework, this study found that BFP was the obesity indicator most strongly associated with autonomic function in Asian young college students, while traditional indicators BMI and WC still provided important incremental information. No statistically significant sex modification was found in the association between obesity and autonomic function, although observed differences in effect sizes suggested potential heterogeneity in susceptibility. We recommend moving beyond BMI in cardiovascular risk screening for youth and integrating body composition measurement into health assessment systems, offering a new approach for early prevention of CVD.

## Author Contributions

Jun Wang (corresponding author): conceptualization, methodology, formal analysis, writing–original draft, and writing–review and editing; Xuechun Ding: conceptualization, investigation, data curation, and validation; Juan Wang: investigation, data curation, and visualization; Juan Wu: conceptualization, investigation, data curation, and validation; Juxia Chen: investigation, data curation, and validation; Dongdong Zhu: investigation, data curation, visualization, and writing–original draft; Zhixiang Peng: investigation, data curation, and visualization.

## Funding

This work was supported by two grants: the 2025 Scientific Research Projects (Natural Science Category) of the Anhui Provincial Department of Education (Wanjiaomike [2025] No. 132, Project No.: 2025AHGXZK30867) and the Cultivation Program for Distinguished Teachers (Master Craftsmen) of Vocational Colleges in Anhui Province for the New Era (2025–2027) (Grant No. Wan Jiao Mi (2025) 281).

## Conflicts of Interest

The authors declare no conflicts of interest.

## Supporting Information

Additional supporting information can be found online in the Supporting Information section.

## Supporting information


**Supporting Information 1** Figure S1. Study population selection flowchart.

**Supporting Information 2** Table S1. Spearman correlation matrices between obesity indices and HRV parameters by sex.

**Supporting Information 3** Table S2. Comparison of AIC values for physical activity covariates across HRV domains.

**Supporting Information 4** Table S3. Univariate analysis results standardized.

**Supporting Information 5** Table S4. Detailed results of the four‐track parallel analysis.

**Supporting Information 6** Table S5. Sensitivity analysis results.

## Data Availability

The datasets used and/or analyzed during the current study are available from the corresponding author on reasonable request.
