# Supplementary figures and images for: Association Between Obesity Indices and Heart Rate Variability: A Cross‐Sectional Study in Asian Young College Students
Source: Cardiol Res Pract. 2026 Jun 13;2026:5718188. doi: 10.1155/crp/5718188 (PMC13263787; doi:10.1155/crp/5718188)

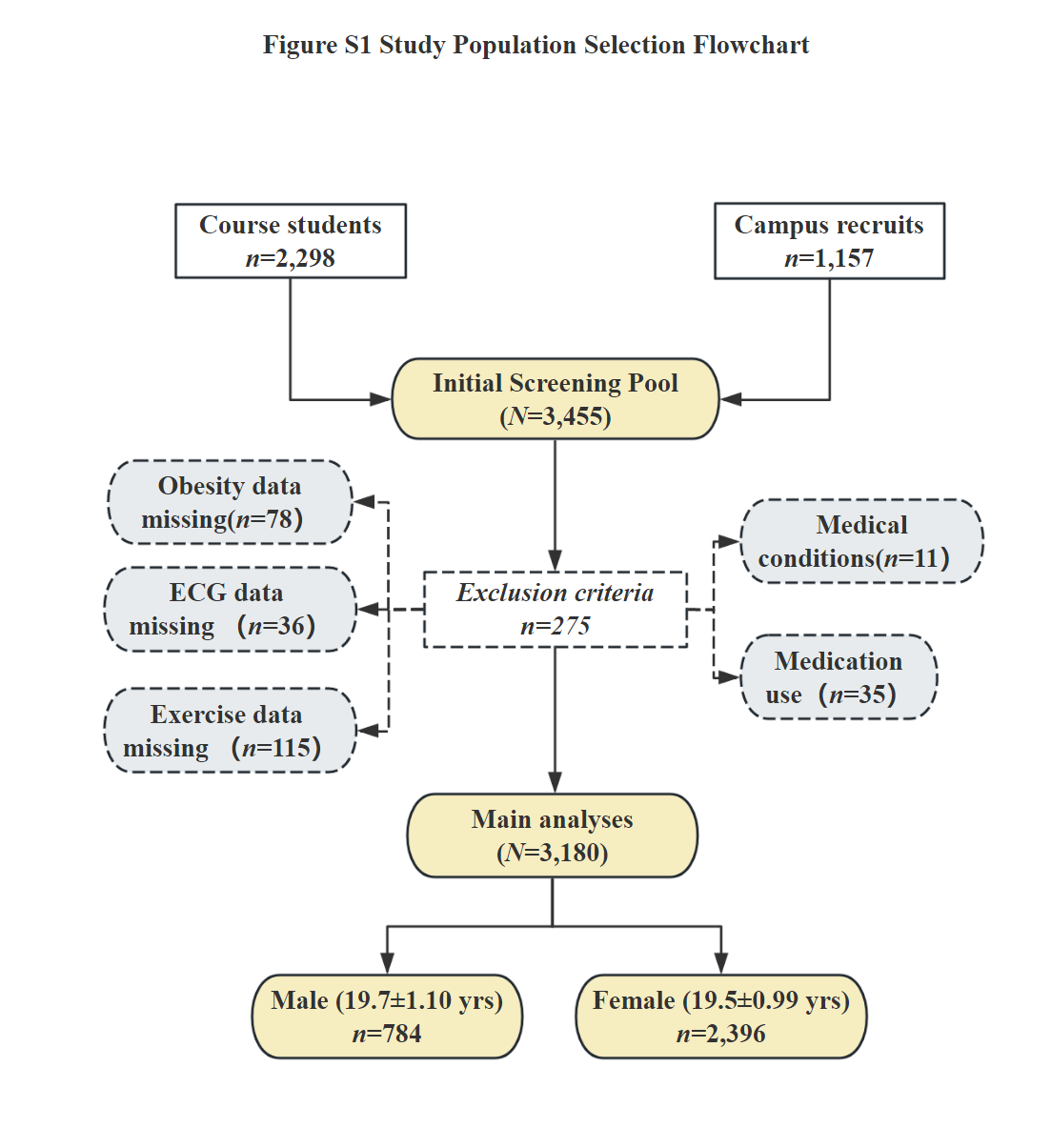

Supplement: Supplementary file 1 — Supporting Information 1 Figure S1. Study population selection flowchart. [file CRP-2026-5718188-s006.tif]
